# Blend of essential oils can reduce diarrheal disorders and improve liver antioxidant status in weaning piglets

**DOI:** 10.5713/ab.22.0072

**Published:** 2022-06-24

**Authors:** Maiara Ananda Grando, Vanessa Costa, Jansller Luiz Genova, Paulo Evaristo Rupolo, Liliana Bury de Azevedo, Leandro Batista Costa, Silvana Teixeira Carvalho, Thiago Pereira Ribeiro, Daniel Pigatto Monteiro, Paulo Levi de Oliveira Carvalho

**Affiliations:** 1 Animal Science, Universidade Estadual do Oeste do Paraná, Marechal Cândido Rondon, 85960-000, Brazil; 2 Animal Science, Universidade Federal de Viçosa, Viçosa, 36570-900, Brazil; 3 Animal Science, Pontifícia Universidade Católica do Paraná, School of Medicine and Life Sciences, Curitiba, 80215-901, Brazil; 4 TECTRON-Tecnologia e Inovação, Toledo, 85908-220, Brazil

**Keywords:** Antimicrobial, Diarrhea Occurrence, Intestinal Histology, Phytogenic Additives, Superoxide Dismutase, Weaning Piglets

## Abstract

**Objective:**

This study was to assess the effects of different doses of an essential oil blend (EOB) on growth performance, diarrhea occurrence (DO), hematological and blood biochemical profile, intestinal morphometry, morphology and microbiology, relative weight and length of organs, digestive content pH, and liver antioxidant status in weaning piglets.

**Methods:**

A total of 135 barrows (7.09±0.29 kg body weight) were allotted randomly in a randomized complete block design based on body weight with nine replications and three animals per pen. Dietary treatments were a negative control (NC): basal diet; positive control (PC): NC plus 125 mg performance-enhancing antibiotic (enramycin 8%)/kg diet; NC plus 100 mg EOB/kg diet (EO100); NC plus 200 mg EOB/kg diet (EO200); and NC plus 400 mg EOB/kg diet (EO400). Diarrhea occurrence was monitored daily, and performance at the end of each phase.

**Results:**

Gain to feed ratio was greater (p<0.05) in starter II pigs fed EO400 and EO200 than in those fed EO100. Pigs fed EO400 had lower (p<0.05) DO than those fed NC and EO100 in the total period. Pre-starter II pigs fed NC had (p<0.05) lower serum total protein and plasma protein than pigs fed PC. Pigs fed EO100 showed smaller (p<0.05) mean corpuscular volume (MCV) than pigs fed EO400. Starter II pigs fed EO400 had (p<0.05) greater MCV and lower mean corpuscular hemoglobin and erythrocytes than those fed EO100. There was a greater concentration (p<0.05) of band cells for PC, similar to EO400 and EO200. Performance-enhancing antibiotic and EOB to diets increased (p<0.05) liver superoxide dismutase activity.

**Conclusion:**

Adding 200 and 400 mg EOB/kg diet decreased DO and was advantageous to hematological and blood biochemical profile and liver antioxidant status without being detrimental to growth performance and gastrointestinal health in nursery pigs.

## INTRODUCTION

Immediate post-weaning is a critical phase for weaning piglets because of the transition from a liquid (milk) to a solid diet while the gastrointestinal tract (GIT) and immune system are immature. Furthermore, other stressing factor such as sow separation, transportation, and new social and environmental interactions can be additional challenges for weaning piglets.

The occurrence of intestinal disorders that affect growth performance is common in weaning piglets. This can be aggravated by the prevalence of pathogenic microorganisms [1]. To alleviate these disorders and optimize animal productivity, sub-therapeutic doses of antimicrobials are added to diets continuously acting as performance-enhancing antibiotics. Conversely, the wide use of performance-enhancing antibiotics (PEA) in animal production coupled with the possible presence of their residues in animal food and in the environment has become a great concern. This is based on the emergence of resistant bacteria with the potential to cause diseases in animals and humans [2].

The use of some antimicrobials as PEA has been banned as food additives throughout the European Union on January 1st, 2006. This has led to the use of other still permitted, such as enramycin, a polypeptide produced during *Streptomyces fungidicus* fermentation. It has antibiotic action against gram-positive bacteria, such as *Clostridium perfringens* [3]. Even so, there is strong pressure for a complete prohibition of PEA use and hence research has focused on studying alternative substances, among which are phytogenic feed additives. These are plant-derived substances that can be classified according to their origin or processing [4].

Essential oils (EO) are phytogenic substances composed of volatile and lipophilic compounds. Essential oils can be extracted from plants through different processes, such as cold expression or steam distillation [4]. These additives have antimicrobial potential due to their ability to change the structure of bacteria cell membranes. Because of their hydrophobic characteristic, EO can associate with proteins present in the membrane and change their functions, affecting cell permeability and resulting in loss of viability. Essential oils are also able to positively affect immune response through modulation of the activity of cellular proteins responsible for releasing inflammatory molecules. Phenolic compounds present in EO can contribute to the body’s antioxidant system through the upregulation of signaling pathways involved in the expression of antioxidant enzymes. Furthermore, EO have been previously reported to increase digestive enzymes secretion in GIT epithelium [5].

Essential oils in their free form when orally administered are rapidly absorbed in GIT (mainly stomach and proximal small intestine). Encapsulated EO can show a slower rate of absorption. Once in the bloodstream, EO by-passe body tissues and most are metabolized by kidneys and excreted in the urine, with a half life of approximately two hours. Thus, there is little possibility of accumulation in animal tissues [6].

There is a wide variety of EO that can be extracted from plants, each with its biological singularity, but few studies have focused on the combinations of more than a couple of isolated compounds. Therefore, we hypothesized that an administration of an EO blend (EOB) (up to 400 mg/kg diet) as an alternative for conventional PEA improves the response of pigs and hence decreases diarrhea, increases liver antioxidant capacity, and growth performance. Based on this line of reasoning, this study was conducted to assess the effects of different doses of EOB on growth performance, diarrhea occurrence (DO), hematological and blood biochemical profile, intestinal morphometry, morphology and microbiology, relative weight and length of organs, digestive content pH, and liver antioxidant status in weaning piglets.

## MATERIALS AND METHODS

The study was conducted in the swine center of the experimental farm (Professor Antônio Carlos dos Santos Pessoa) belonging to Unioeste, Brazil.

### Animal care

All animal procedures were approved by the Ethics Committee on the Use of Animals in Experimentation at the Universidade Estadual do Oeste do Paraná - Unioeste (protocol no. 02/2020-CEUAP).

### Animals, experimental design, and housing

A total of 135 crossbreed barrow piglets (Landrace×Large White, Agroceres♂ and DanBred♀) weaned at 25 d were used. Initial body weight (BW) was 7.09±0.29 kg. Pigs were allotted randomly to one of the five treatments in a randomized complete block design with three replications in each of three batches over time resulting in a total to nine replicates per treatment and three animals per pen.

Pigs were weighed, identified with an ear tag, and housed in a masonry nursery room with raised slatted plastic floor pens (1.5 m^2^) equipped with gutter feeders and nipple drinkers. Pens were washed daily (in the afternoon) with running water. The experiment lasted 42 d.

### Experimental diets

Dietary treatments were as follows: negative control (NC): basal diet; positive control (PC): negative control plus 125 mg PEA (enramycin 8%)/kg diet; negative control plus 100 mg EOB/kg diet (EO100); negative control plus 200 mg EOB/kg diet (EO200); and negative control plus 400 mg EOB/kg diet (EO400; Table 1).

The EOB was a non-encapsulated commercially available product (Tectron, Toledo, PR, Brazil) containing 10% thymol, 10% cinnamaldehyde, 10% d-limonene, 7.5% carvacrol, and 62.5% rice bran. Performance-enhancing antibiotic and EOB were added to diets replacing ground corn to keep similar nutritional composition among treatments.

All diets were corn and soybean meal-based and were offered as mash. Diets were divided into four phases (pre-starter I, pre-starter II, starter I, and starter II) and formulated taking into account the nutritional requirements limits proposed by Rostagno et al [7]. Pigs were allowed *ad libitum* access to feed and water throughout the experiment.

Samples of basal diets were analyzed (Table 2) for moisture (method 53), ether extract (method 12), crude fiber (method 18), acid detergent fiber (method 19), neutral detergent fiber (method 20), ash content (method 05), total lactose (method 24), crude protein (method 47), total calcium (method 04), and total phosphorus (method 23), as previously described by Nogueira et al [8].

### Growth performance and diarrhea occurrence

The average final BW (kg), the average daily gain (ADG, g/d), the average daily feed intake (ADFI, g/d), and gain to feed ratio (G:F, g/g) were evaluated to address growth performance. Pigs were weighed (UL-50 Digital Scale; DIGI-TRON, Curitiba, Brazil) at the beginning and the end of each phase, as well as the feed offered and leftovers. Average daily gain was determined by the difference between initial and final BW divided by the days of each phase. Average daily feed intake was calculated by subtracting the leftovers from the amount of feed offered in each phase. Gain to feed ratio was calculated by dividing ADG by ADFI.

Diarrhea occurrence was observed throughout the experiment. Feces of animals from each treatment were scored daily (1000 h) on a 4-point scale (0 = formed feces, 1 = soft feces, 2 = semi-solid feces, and 3 = liquid feces) as previously described by Huang et al [9]. Data on fecal scores were transformed into binary traits as follows: 0 = no diarrhea (fecal scores = 0 and 1) and 1 = presence of diarrhea (fecal scores = 2 and 3). The frequency of DO in each phase was calculated by dividing the sum of DO by the total number of observations in each treatment and then multiplying by 100%. Results were expressed as observed percent (%).

### Hematological and blood biochemical profile

At the end of pre-starter II and starter II, 18 pigs per treatment were selected for blood collection based on the closest BW relative to the average BW in each experimental unit.

Pigs fasted for 10 h and then blood samples (≅ 20 mL) were withdrawn from vena cava (0800 h) using 0.70×30 mm needles. Blood was collected in tubes containing ethylenediaminetetraacetic acid for determination of complete blood count, potassium fluoride for determination of glucose (GLU), heparin for determination of urea (URE), alanine aminotransferase (ALT), and alkaline phosphatase (AP). Tubes with no anticoagulant were used for the determination of albumin (ALB) and total protein (TP). Blood samples were placed on ice inside of a cooler (4°C) to be sent to the laboratory.

The complete blood count was determined in the laboratory of the veterinary hospital at Universidade Federal do Paraná (Palotina, PR, Brazil) using an automated hematology analyzer (BS 120; Mindray, Shenzhen, China). Erythrocytes (ER), hemoglobin (HG), hematocrit (HT), total leucocytes, segmented neutrophils, band cells, eosinophils, basophils, lymphocytes, monocytes, platelets, plasma protein (PP), mean corpuscular volume (MCV), mean corpuscular hemoglobin (MCH), and mean corpuscular hemoglobin concentration were determined.

Samples for biochemical evaluation were centrifuged at 3,000 *g* (80-2B; Centrilab, Hangzhou, China) for 10 minutes at the blood analysis laboratory of Unioeste. Then, approximately 3 mL of supernatants were collected and duplicates were stored in microtubes at −20°C. Albumin and TP were analyzed via colorimetry, ALT via the kinetic method, and AP, URE, and GLU via enzymatic-colorimetry, using commercial kits (Gold Analisa Diagnóstica Ltda., Belo Horizonte, Brazil) and a Bel SPECTRO S05 spectrophotometer (Bel Engineering, Monza, Italy).

### Intestinal microbiology

At the end of the experimental period (42 d), six piglets per treatment were slaughtered for GIT sampling. Pigs were selected based on the closest BW relative to the final average BW of all replicates. Pigs were slaughtered in a commercial abattoir after fasting for approximately 10 hours. All euthanasia procedures were performed by electronarcosis following Brazilian guidelines (Resolution No. 37 of February 15, 2018, CONCEA).

Samples of jejunum, ileum, and colon contents were collected and individually placed in sterile plastic containers. Then, at the microbiology lab from Unioeste, 1 g of each sample was serially diluted in 1% peptone water. Each dilution was vortexed (K45-2810; KASVI, Taizhou, China) for 30 seconds. A 100 μL aliquot of each diluted sample was spread evenly on the surface of eosin-methylene blue agar plates for Enterobacteriaceae (ETB) counting. A 1 mL of diluted sample was inoculated on tryptose-sulfite-cycloserine and de Man, Rogosa and Sharpe agar plates for sulfite-reducing clostridia (SRC) and lactic acid bacteria (LAB) counting, respectively.

Then, plates were incubated at 37ºC (EL 202; Eletrolab, São Paulo, Brazil) aerobically for 24 h (ETB) and anaerobically for 48 h (SRC and LAB) as previously described by Da Silva et al [10]. After incubation, bacterial colonies were counted and data were multiplied by the respective dilution and log-transformed (Log_10_). Results were expressed in log colony forming unit/g.

### Intestinal morphometry and morphology

Samples (≅ 3 cm) of jejunum (150 cm cranial to ileocecal junction), ileum (15 cm cranial to ileocecal junction), and colon (100 cm caudal to ileocecal junction) were taken. Fragments collected were washed with saline (0.9% sodium chloride) and stored in sterilized plastic containers with a 10% buffered formalin solution.

Samples were sent to a commercial laboratory (Mercolab, Cascavel, PR, Brazil) where they were processed in paraffin, and stained with hematoxylin and eosin to prepare slides as previously reported by Prophet et al [11]. A total of 10 villus height (VH) and their crypt depth (CD) were measured. Then, VH to CD ratio was calculated as previously described by Kisielinski et al [12]. Intestinal morphology was also assessed via infiltrate, congestion, desquamation, coccidiosis, bacterial lumps, band cells, cysts, mucus, necrosis, and edema observations as previously described by Kraieski et al [13]. Histological analyses were performed using an optical microscope (CX31RTSF; Olympus, Tokyo, Japan) and a computer system (ToupView ×86).

### Relative weight and length of organs and pH of the digestive contents

After slaughter, the contents of the stomach, jejunum, ileum, cecum, and colon of the pigs were sampled and stored in plastic containers. Then, pH was measured using a digital pH meter (TEC-2 mp; TECNAL, Piracicaba, Brazil).

The liver and gallbladder, heart, spleen, kidneys, empty stomach, small intestine (duodenum, jejunum, and ileum), and large intestine (cecum, colon, and rectum) were weighed using a digital scale (MX-111; Maxon, Beijing, China). Relative organ weight to final BW was then calculated. Small and large intestine length was measured using a measuring tape.

### Liver antioxidant status

Liver samples (≅ 2 g) were collected in microtubes and immediately placed in cold chamber at −20°C for further storage in ultrafreezer (CL580-86V; ColdLab, Piracicaba, Brazil) at −80°C. Samples were then shipped to a commercial laboratory (Imunova, Curitiba, Brazil) in a cooler with solid carbon dioxide (−60°C) to be analyzed for glutathione peptide (GSH), liver protein (LP), and enzymes: glutathione S-transferase (GST), superoxide dismutase (SOD), and catalase (CAT).

Liver samples were homogenized in potassium phosphate buffer solution (pH 6.5) with 10-fold dilution for GSH, SOD, CAT, and LP analyses, or 30-fold dilution for GST analysis. Samples were then centrifuged (NT 805; Nova Técnica, Piracicaba, Brazil) at 10,000 g at 4°C for 20 min.

The activity of CAT was measured as previously described by Aebi [14]. Briefly, the reaction was performed using 5 mM hydrogen peroxide and 50 mM phosphate buffer (pH 7.0) solution with the cytosolic protein. The reaction was continuously monitored at 240 nm for 60 sec using a microplate reader (Synergy HT; Biotek, Winooski, VT, USA). The extinction coefficient was 41 mM/cm.

Superoxide dismutase activity was determined based on inhibition of the autoxidation of pyrogallol [15]. Briefly, samples (60 μL) were diluted in 1,327.5 μL Tris-HCl buffer (0.4 M, pH 8.9) and vortexed (QL-901; Biomixer, Rancho Cucamonga, CA, USA). Then, 75 μL of pyrogallol (15 mM) were added to diluted samples and incubated for 30 min at room temperature. The reaction was stopped by adding 37.5 μL HCl (1 N) and then absorbance was measured at 440 nm using the microplate reader. The amount of enzyme that inhibited the reaction by 50% was defined as one unit of SOD. The enzyme activity was expressed as SOD unities per milligram of total LP (SOD U/mg of LP).

Glutathione S-transferase analysis was performed by adding 200 μL of a solution (containing 3 mM 1-chloro-2,4-dinitrobenzene (CDNB), diluted in ethanol, and 3 mM GSH, diluted in potassium phosphate buffer) to 100 μL of sample supernatant. The linear increase of absorbance at 340 nm was monitored using an extinction coefficient of 9.6 mmolar/cm. Glutathione S-transferase catalyzes the conjugation of CDNB to GSH (reduced glutathione). The product of this reaction is a thioether that can be monitored by the increase in absorbance, as previously described by Habig et al [16].

Glutathione peptide concentration was measured by the method of Sedlak and Lindsay [17]. Briefly, 80 μL of trichloroacetic acid (12.5%) was added to 100 μL of the sample homogenate and centrifuged at 6,000 *g* at 4°C for 15 min. Then, 20 μL of the supernatant was mixed with 280 μL of Tris-HCl buffer (0.4 M, pH 8.9) and 5 μL of 5,5′-dithiobi-2-nitrobenzoic acid in methanol. Absorbance was measured at 415 nm using the microplate reader. A known GSH concentration solution was used as an external standard.

Liver protein was determined using bovine ALB as a standard as previously described by Bradford [18]. Briefly, 10 μL of the sample was added to 250 μL of Bradford solution in each microplate well. Absorbance was measured at 595 nm using the microplate reader. The LP results were used to calculate the previous liver variables and were expressed as mg of protein in liver homogenates.

### Statistical analysis

Statistical analyzes were performed using the SAS University Edition software (SAS Inst. Inc., Cary, NC, USA). Residual error was evaluated for outliers based on the normal distribution curve via the Student test (RStudent). If studentized residuals met or exceeded three standard deviations, the sample was considered significant.

The normality of experimental errors and the homogeneity of variances among treatments were evaluated using Shapiro-Wilk and Levene tests, respectively. The non-influence (p>0.05) of treatments on the initial BW of the piglets, for indication as a covariate and correction of observed average values, was verified via analysis of variance (ANOVA).

Data on growth performance, blood biochemical profile, intestinal morphometry and microbiology, liver antioxidant status, relative weight and length of organs, and pH of the digestive content were analyzed via analysis of covariance or ANOVA. The model included treatment as a fixed effect and block and residual error as a random effect with an individual pen as the experimental unit. The factors included in the model used were Y_ijk_ = μ+T_i_+b_j_+β (X̄_ijk_...)+ɛ_ijk_, in which Y_ijk_ = average observation of the dependent variable in each plot, measured in the i-th treatment, in the j-th block and in the k-th replication; μ = effect of the overall average; T_i_ = effect of class of treatment, for i = (1, 2, 3, 4, and 5); b_j_ = effect of block classes, for j = (1, 2, and 3); β = regression coefficient of Y about X; X_ijk_ = average observation of the covariate (initial BW) in each plot, measured in i-th treatment class, in j-th block class and in k-th replication; X̄... = overall average for the covariate X; ɛ_ijk_ = random error of the plot associated with level i, block j, and replication k.

A generalized linear model was fitted for each distribution and linkage function to analyze data on DO, intestinal morphology, and hematological profile. The treatment effect was verified via type III analysis. The Akaike information criteria was used to test the model fitting. Generalized linear model used was represented by the systematic portion: η = μ+T_i_+b_j_, wherein μ was the effect associated with the overall average; T_i_ was the effect associated with i-th treatment class, for i = (1, 2, 3, 4, and 5) and b_j_ was the effect associated with j-th block, for j = (1, 2, and 3).

The Student-Newman-Keuls test was used to compare pairs of treatment averages for growth performance, blood biochemical profile, intestinal morphometry and microbiology, liver antioxidant status, relative weight and length of organs, and pH of the digestive content. The Tukey-Kramer test was used to compare the averages for DO, intestinal morphology, and hematological profile. Significant differences were set at p<0.05. Results were reported as means with pooled standard error of the mean.

## RESULTS

### Growth performance and diarrhea occurrence

There was no effect of treatment on growth performance in pre-starter I, II, and starter I phases (Table 3). Starter II pigs fed EO400 and EO200 showed (p<0.05) greater G:F than those fed EO100. Pre-starter I pigs fed EO400 and NC had lower (p<0.05) DO than those fed EO100. In pre-starter II, piglets consuming EO400 exhibited (p<0.05) a reduction in DO than those on NC, EO100, and EO200. There was no effect of treatments on the DO in starter I phase. Starter II pigs fed EO400 showed (p<0.05) lower DO than those fed NC and EO100. A similar result was observed in the total experimental period.

### Hematological and blood biochemical profile

Lower TP was observed (p<0.05) in pre-starter II pigs fed NC (Table 4). Pre-starter II pigs fed EO400 showed (p<0.05) greater MCV than pigs fed EO100 and NC. Pre-starter II pigs fed PC showed (p<0.05) greater PP than those fed NC. At the end of starter II, greater MCV (p<0.05) and lower ER (p<0.05) and MCH (p<0.05) were observed in pigs fed EO400 when compared to those fed EO100. Band cells concentration was greater (p<0.05) in pigs fed PC, with results similar to those fed EO200 and EO400.

### Intestinal microbiology, morphometry and morphology

No treatment effect was observed on colony-forming units of ETB, SRC, and LAB in the different intestinal segments of the pigs (Table 5). There was no effect on intestinal morphometry and morphology in pigs (Table 6).

### Relative weight and length of organs and pH of the digestive contents

There was no treatment effect on relative weight and length of organs and pH of digestive content in pigs (Table 7).

### Liver antioxidant status

Pigs fed NC showed (p<0.05) lower hepatic SOD activity (Table 8).

## DISCUSSION

### Growth performance and diarrhea occurrence

In general, the different treatments did not affect growth performance in the present study. Kommera et al [19] suggested that a more controlled environment, such as experimental units, may affect the animals’ response to the use of growth-promoting feed additives because there are fewer stressing factors and strict sanitation. The composition of the diets may have contributed to the results as well. It is worth mentioning that the nutritional support provided by the diets, associated with the absence of challenges, did not allow the tested additives to positively influence pig performance.

Weaning is characterized by environmental, social, and dietary changes which can negatively impact on the intestinal health and growth of nursery pigs and hence favoring DO [1]. In this study, supplementation of EOB to the diets, at the highest experimental dose, reduced DO. Similarly, Tian and Piao [20] observed lower DO in pigs fed 100 mg/kg of a feed additive containing thymol and cinnamaldehyde, and associated the results with small intestine morphology improvement. However, it was not observed in the present study, therefore we relate the antidiarrheal effect of the EOB to a possible GIT spasmolytic activity, mainly caused by cinnamaldehyde.

Diarrhea can be defined as the excretion of liquid feces along with water and electrolytes from the GIT which are expelled through abnormal intestinal muscle contractions. It can be associated or not with microorganism infections [21]. Cinnamaldehyde has been previously reported to decrease gastrointestinal motility in other species by blocking calcium channels, which are involved in muscle contractility, and by inducing adrenaline secretion, another spasmolytic agent [21,22].

### Hematological and blood biochemical profile

Our observations suggest that DO may be related to the blood tests results because pigs fed EO100 showed higher ER and MCH, and lower MCV along with higher DO. Erythrocytes, MCV, and MCH are the number of red cells present in the sample, their volume, and the amount of hemoglobin contained in each one of them, respectively. In cases of dehydration, there may be a higher concentration of red cells in the sample due to a lower water concentration in blood. Thus, there may be an increase in hemoglobin in the sample, which has affected MCH, and a decrease in MCV because red cells with less intracellular water have a smaller volume [23].

Blood analysis also showed a treatment effect on TP and PP in pre-starter II pigs. Total protein analysis method differs from PP regarding its absence of fibrinogen in the sample. However, both analyses count the number of ALBs and globulins in the blood. Higher TP values coupled with no change in ALB may be related to an increase in the number of globulins [24]. Huang and Lee [25] mention that EO such as carvacrol, cinnamaldehyde and thymol, are able to increase blood levels of antibodies (e.g. globulins), and it is believed to be through the modulation of mitogen-activated protein kinases and nuclear factor kappa B signaling, the two major pathways involved in immune responses. Regarding PP, globulins, and fibrinogen content in each sample may be associated with the results we observed.

In starter II, there was a treatment effect on band cells which are neutrophils not fully developed and are part of the innate immune system. These cells play role in inflammatory responses to infections or tissue injuries. They are released into the bloodstream when the bone marrow is stimulated by pro-inflammatory substances [26]. Schepetkin et al [27] reported that EO can modulate neutrophils migration, thus improving the immune response. This explains the higher amount of band cells in pigs fed EOB.

### Intestinal microbiology, morphometry and morphology

Essential oils can improve intestinal epithelium and modulate its microbiota via an antimicrobial effect on pathogenic species [5]. In the study conducted by Wei et al [28], a lower count of *Escherichia coli* and a higher count of *Lactobacillus* in the jejunum of pigs fed a blend of 50 mg thymol/kg and 50 mg carvacrol/kg were observed. Tian and Piao [20] observed a higher duodenum VH and jejunal VH to CD ratio in pigs (8.1±1.4 kg BW) fed 100 mg thymol and cinnamaldehyde/kg when compared to pigs fed a control diet. However, no effect of treatments on intestinal morphometry, morphology, and microbiology was observed in the present study. A possible explanation for this is that EOB compounds were not microencapsulated. This is corroborated by Michiels et al [6] who reported that non-encapsulated compounds, such as carvacrol and thymol, are mostly absorbed in the stomach and hence less absorbed in the intestine. Furthermore, as they are rapidly absorbed by GIT epithelium there is little time to affect microbiota in the intestinal lumen.

### Relative weight and length of organs and pH of the digestive contents

No treatment effect was observed for the relative weight and length of organs and pH of digestive contents. This concurs with the findings of Gois et al [29] and Cairo et al [30] who studied different doses (500; 1,000; 1,500 mg/kg) of *Schinus terebinthifolius* Raddi EO in pigs (5.65±0.78 kg BW) weaned at 21 d. These results suggest that EO can be used in pig feeding without detrimental effects on organs.

### Liver antioxidant status

Different stressing factors, such as the weaning period and pathogen infections, can increase the production of reactive oxygen species (ROS) in vital tissues, and hence make pigs more susceptible to cell injury. Reactive oxygen species are highly reactive metabolites that result from the normal cellular energy metabolism in mitochondria where oxygen is an electron acceptor for enzyme reactions [31]. To reduce ROS oxidative damage, cells are equipped with an antioxidant system formed by enzymatic compounds (e.g. SOD, CAT, and GST) and non-enzymatic compounds (e.g. GSH). Oxidative stress occurs when ROS concentration exceeds the capacity of action of antioxidant agents [32]. For this reason, the activities of these enzymes in animal tissues have been used as indicators of oxidative stress [33]. In the present study, an effect of the treatments on liver SOD activity was observed. According to Asakura and Kitahora [34], during cellular metabolic reactions, the first produced ROS is the superoxide anion (O_2_^−^). Superoxide dismutase converts this anion into hydrogen peroxide (H_2_O_2_), which is subsequently decomposed to water (H_2_O) by the actions of CAT and glutathione peroxidase. The tripeptide GSH also participates in these reactions as oxygen acceptor (from H_2_O_2_).

The increased SOD activity we observed with EOB supplementation was considered beneficial, as there was no reduction in GSH levels. This suggests EOB was able to stimulate the antioxidant defense system without increasing the concentration of ROS. Otherwise, there would be a lower concentration of GSH due to its participation in H_2_O_2_ neutralization reactions.

Zou et al [32] reported that the possible mechanism by which some EO can increase SOD activity is through the activation of the nuclear factor erythroid 2-related factor 2 (Nrf2). This intracellular protein (not evaluated in the present study) regulates the expression of genes encoding antioxidant enzymes. In the study conducted by Tian and Piao [20], an increase in serum SOD and CAT activities was observed in pigs (8.1±1.4 kg BW) supplemented with 100 mg thymol and cinnamaldehyde/kg. Furthermore, increased SOD activity was reported in the liver and jejunum of the animals, with results similar to those of the treatment containing PEA.

Previous studies related to the use of EO have shown varied results on the effects of these compounds on animal organisms, as evidenced in the present study. Ahsan et al [35] suggested different doses of EO may be necessary to influence different parameters. This highlights the importance of studying these compounds to understand their effectiveness in the nutrition of weaned pigs.

## CONCLUSION

In conclusion, supplementation 200 and 400 mg EOB/kg diet decreased DO and was advantageous to hematological and blood biochemical profile and liver antioxidant status. In addition, different doses of EOB and antibiotic did not positively affect growth performance or gastrointestinal parameters, but did not impair the development of the animals. Thus, EOB can be a promising alternative for maintaining health in nursery pigs.

## Figures and Tables

**Table 1:** Ingredients of experimental diets for weaning piglets (as-fed basis)

Ingredients	Basal diets (g/kg)			

	Pre-starter I	Pre-starter II	Starter I	Starter II
Corn (7.6% crude protein)	355.0	435.0	500.0	655.0
Pregelatinized corn (8.0% crude protein)	164.1	121.1	65.4	11.9
Soybean meal (46% crude protein)	120.0	140.0	180.0	225.0
Micronized soybean meal (39% crude protein)	50.0	40.0	30.0	20.0
Fish meal (65% crude protein)	30.0	30.0	30.0	30.0
Soybean oil	25.0	25.0	20.0	20.0
Milk powder	115.0	85.0	80.0	-
Dried whey	100.0	85.0	60.0	-
Monosodium glutamate	0.1	0.1	0.1	0.1
Sugar	10.0	10.0	10.0	10.0
Salt	5.0	5.0	5.0	5.0
Calcitic limestone	3.0	3.4	2.3	3.9
Dicalcium phosphate	6.9	5.2	5.3	6.9
L-lysine HCl (80%)	5.6	5.4	3.9	4.4
DL-methionine (98%)	2.6	2.4	1.5	1.4
L-threonine (98%)	2.9	2.8	2.3	2.4
L-tryptophan (98%)	0.9	0.9	0.6	0.6
L-valine (98%)	0.5	0.4	0.3	0.2
Mycotoxin binder	1.0	1.0	1.0	1.0
Mineral premix^1)^	0.5	0.5	0.5	0.5
Vitamin premix^2)^	0.2	0.2	0.2	0.2
Choline chloride (60% content)	0.5	0.5	0.5	0.5
Copper sulfate (35% content)	0.3	0.3	0.3	0.3
BHT	0.2	0.2	0.2	0.2
Phytase^3)^	0.1	0.1	0.1	0.1

BHT, butylated hydroxytoluene.

1) Mineral premix (provided per kg of premix): zinc (zinc oxide), 160 g; manganese (manganese monoxide), 120 g; iron (iron sulfate), 120 g; copper (copper sulfate), 20 g; iodine (calcium iodate), 2,000 mg.

2) Vitamin premix (provided per kg of premix): vitamin A, 28,800 KIU; vitamin D_3_, 6,400 KIU; vitamin E, 78,933 IU; vitamin K_3_, 12.80 g; vitamin B_1_, 6,400 mg; vitamin B_2_, 16 g; vitamin B_12_, 64,000 μg; niacin, 128 g; pantothenic acid, 64 g; folic acid, 1,980 mg; biotin, 640 mg; selenium (sodium selenite), 1,200 mg.

3) Phytase, 500 FTU/kg.

**Table 2:** Nutrient composition of experimental diets for weaning piglets (as-fed basis)

Items	Basal diets			

	Pre-starter I	Pre-starter II	Starter I	Starter II
Calculated values (%)				
Metabolizable energy (kcal/kg)	3,600	3,550	3,500	3,450
SID lysine	1.35	1.30	1.25	1.20
SID methionine+cystine	0.78	0.75	0.70	0.67
SID threonine	0.84	0.82	0.79	0.78
SID tryptophan	0.27	0.26	0.25	0.24
SID valine	0.63	0.64	0.68	0.73
Analyzed values (%)				
Crude protein	18.95	18.76	19.46	19.30
Ether extract	6.73	7.99	6.87	5.84
Crude fiber	1.73	1.67	1.99	2.16
ADF	2.28	3.32	4.40	2.96
NDF	6.89	6.56	8.47	8.99
Ash content	4.65	4.37	4.34	4.23
Total calcium	0.68	0.68	0.63	0.70
Total phosphorus	0.58	0.52	0.55	0.52
Lactose	12.70	10.81	7.57	-

SID, standardized ileal digestible; ADF, acid detergent fiber; NDF, neutral detergent fiber.

**Table 3:** Effect of an essential oil blend on growth performance and diarrhea occurrence in weaning piglets

Item	Treatments^1)^					SEM	p-value

	NC	PC	EO100	EO200	EO400		
Pre-starter I (14 days of experimentation)							
IBW (kg)	7.06	7.10	7.10	7.10	7.10	-	-
FBW (kg)	9.48	9.74	9.58	9.33	9.85	0.096	0.287
ADG (g)	169	185	174	159	192	0.005	0.361
ADFI (g)	259	281	264	246	275	0.006	0.359
G:F	0.65	0.66	0.66	0.65	0.70	0.012	0.638
DO (%)^2)^	75.19^b^	77.52^ab^	88.37^a^	79.84^ab^	73.64^b^	-	0.013
Pre-starter II (25 days of experimentation)							
FBW (kg)	13.83	14.51	14.20	13.68	14.52	0.160	0.664
ADG (g)	394	434	420	398	427	0.008	0.659
ADFI (g)	563	605	586	565	587	0.010	0.866
G:F	0.70	0.72	0.72	0.70	0.73	0.006	0.448
DO (%)^2)^	52.53^a^	42.42^ab^	55.56^a^	52.53^a^	37.37^b^	-	0.041
Starter I (33 days of experimentation)							
FBW (kg)	18.73	19.72	18.98	18.45	19.36	0.187	0.539
ADG (g)	642	661	631	616	634	0.011	0.602
ADFI (g)	884	937	901	868	884	0.014	0.502
G:F	0.73	0.70	0.70	0.71	0.72	0.006	0.548
DO (%)^2)^	36.23	26.09	28.99	31.88	40.58	-	0.351
Starter II (42 days of experimentation)							
FBW (kg)	23.54	24.14	23.62	23.43	24.41	0.231	0.129
ADG (g)	627	622	610	654	664	0.009	0.081
ADFI (g)	1,004	1,026	1,033	1,020	1,028	0.014	0.601
G:F	0.63^AB^	0.61^AB^	0.59^B^	0.64^A^	0.65^A^	0.007	0.047
DO (%)^2)^	60.87^ab^	42.03^abc^	63.77^a^	39.13^bc^	31.88^c^	-	<0.001
Total period (42 days of experimentation)							
FBW (kg)	23.54	24.14	23.62	23.43	24.41	0.230	0.578
ADG (g)	405	419	406	402	426	0.005	0.575
ADFI (g)	599	625	614	587	614	0.008	0.676
G:F	0.68	0.67	0.66	0.68	0.70	0.004	0.124
DO (%)^2)^	59.02^ab^	51.64^bc^	63.66^a^	55.74^abc^	49.73^c^	-	<0.001

SEM, standard error of the mean; IBW, initial body weight; FBW, final body weight; ADG, average daily gain; ADFI, average daily feed intake; G:F, gain to feed ratio; DO, diarrhea occurrence.

1) NC, negative control: basal diet; PC, positive control: NC+125 mg performance-enhancing antibiotic (enramycin 8%)/kg diet; EO100, NC+100 mg essential oil blend/kg diet; EO200, NC+200 mg essential oil blend/kg diet; EO400, NC+400 mg essential oil blend/kg diet.

2) DO (%) = dividing the sum of DO by the total number of observations in each treatment and then multiplying by 100%.

A,B Averages with different uppercase superscripts in the same row differ significantly by the Student-Newman-Keuls test (p<0.05).

a–c Averages with different lowercase superscripts in the same row differ significantly by the Tukey-Kramer test (p<0.05).

**Table 4:** Effect of an essential oil blend on the hematological and blood biochemical profile of weaning piglets

Item	Treatments^1)^					SEM	p-value

	NC	PC	EO100	EO200	EO400		
Pre-starter II (25 days of experimentation)							
ALB (g/dL)	1.87	1.81	1.74	1.84	1.80	0.055	0.718
ALT (U/L)	67.86	59.59	62.87	53.72	64.42	3.729	0.202
AP (U/L)	276.40	276.04	302.48	282.89	267.12	7.660	0.630
GLU (mg/dL)	109.63	120.27	112.15	116.97	113.94	3.366	0.820
TP (g/dL)	3.96^B^	4.76^A^	4.88^A^	4.97^A^	4.77^A^	0.186	0.004
URE (mg/dL)	10.98	8.91	9.47	10.60	9.89	0.384	0.241
ER (10^6^/mm^3^)	5.97	5.96	6.04	6.25	5.92	0.060	0.419
HG (g/dL)	10.03	10.05	9.91	10.51	10.37	0.117	0.314
HT (%)	30.59	31.44	30.56	32.41	32.00	0.283	0.105
MCV (fL)	51.29^b^	53.01^ab^	50.70^b^	51.97^ab^	54.19^a^	0.345	0.007
MCH (pg)	19.58	18.98	19.58	20.26	19.18	0.234	0.393
MCHC (g/dL)	32.78	31.93	32.42	32.42	32.39	0.210	0.776
PP (g/dL)	5.05^b^	5.43^a^	5.30^ab^	5.18^ab^	5.23^ab^	0.038	0.026
Platelets (10^3^/mm^3^)	716.18	644.25	643.06	695.53	662.67	17.909	0.652
Leucocytes (mm^3^)	16,158	16,575	15,918	16,464	17,333	-	0.824
Band cells (%)	0.00	1.63	0.00	0.00	0.00	-	0.160
Seg. neutrophils (%)	43.59	33.50	41.88	44.47	42.72	1.384	0.097
Eosinophils (%)	1.47	1.38	1.31	1.00	1.22	-	0.831
Basophils (%)	0.18	4.44	0.13	0.12	0.00	-	0.303
Lymphocytes (%)	51.76	56.56	53.81	52.00	53.94	1.415	0.843
Monocytes (%)	3.00	2.50	2.88	2.41	2.11	-	0.658
Starter II (42 days of experimentation)							
ALB (g/dL)	1.89	2.03	1.87	2.05	1.77	0.046	0.071
ALT (U/L)	61.21	64.00	55.05	56.05	59.46	2.491	0.613
AP (U/L)	374.28	361.14	381.32	389.84	365.81	9.478	0.880
GLU (mg/dL)	106.79	105.63	108.96	109.26	104.57	2.699	0.803
TP (g/dL)	5.26	5.24	5.36	5.08	4.93	0.114	0.431
URE (mg/dL)	16.07	13.08	14.66	15.33	15.97	0.458	0.176
ER (10^6^/mm^3^)	6.36^ab^	6.52^ab^	6.71^a^	6.59^ab^	6.31^b^	0.044	0.015
HG (g/dL)	11.10	11.45	11.39	11.58	11.18	0.075	0.284
HT (%)	34.50	34.94	35.00	35.71	34.67	0.208	0.521
MCV (fL)	54.24^ab^	53.60^ab^	52.27^b^	54.30^ab^	55.09^a^	0.307	0.013
MCH (pg)	20.47^ab^	21.38^ab^	21.84^a^	21.36^ab^	20.36^b^	0.165	0.011
MCHC (g/dL)	32.19	32.78	32.55	32.43	32.26	0.110	0.425
PP (g/dL)	5.31	5.48	5.49	5.44	5.36	0.043	0.345
Platelets (10^3^/mm^3^)	570.50	519.65	565.41	546.36	527.67	12.007	0.574
Leucocytes (mm^3^)	15,000	14,864	14,170	13,621	14,022	-	0.528
Band cells (%)	0.00^c^	0.24^a^	0.06^cb^	0.21^ab^	0.22^ab^	-	0.004
Seg. neutrophils (%)	39.19	35.88	36.76	39.79	36.72	0.947	0.594
Eosinophils (%)	1.25	1.41	1.41	1.79	0.78	-	0.149
Basophils (%)	3.56	0.00	0.00	0.07	0.00	-	0.350
Lymphocytes (%)	52.25	58.76	57.41	52.43	59.39	1.174	0.142
Monocytes (%)	3.75	3.71	4.35	3.64	2.89	-	0.378

SEM, standard error of the mean; ALB, albumin; ALT, alanine aminotransferase; AP, alkaline phosphatase; GLU, glucose; TP, total protein; URE, urea; ER, erythrocytes; HG, hemoglobin; HT, hematocrit; MCV, mean corpuscular volume; MCH, mean corpuscular hemoglobin; MCHC, mean corpuscular hemoglobin concentration; PP, plasma protein; Seg. neutrophils, segmented neutrophils.

1) NC, negative control: basal diet; PC, positive control: NC+125 mg performance-enhancing antibiotic (enramycin 8%)/kg diet; EO100, NC+100 mg essential oil blend/kg diet; EO200, NC+200 mg essential oil blend/kg diet; EO400, NC+400 mg essential oil blend/kg diet.

A,B Averages with different uppercase superscripts in the same row differ significantly by the Student-Newman-Keuls test (p<0.05).

a–c Averages with different lowercase superscripts in the same row differ significantly by the Tukey-Kramer test (p<0.05).

**Table 5:** Effect of an essential oil blend on intestinal microbiology (log_10_ colony forming unit/g) of weaning piglets at 42 days of experiment

Item	Treatments^1)^					SEM	p-value

	NC	PC	EO100	EO200	EO400		
Jejunum							
Enterobacteriaceae	6.83	6.91	6.39	6.74	5.59	0.280	0.678
Sulfite-reducing clostridia	7.01	6.23	6.18	6.65	6.23	0.166	0.587
Lactic acid bacteria	7.81	7.86	8.08	7.28	7.67	0.167	0.532
Ileum							
Enterobacteriaceae	6.97	7.62	7.42	6.65	5.51	0.265	0.160
Sulfite-reducing clostridia	7.21	6.88	6.80	7.10	6.37	0.207	0.706
Lactic acid bacteria	8.26	8.10	8.32	8.03	7.58	0.149	0.586
Colon							
Enterobacteriaceae	6.25	7.25	5.45	6.69	6.03	0.261	0.472
Sulfite-reducing clostridia	7.08	6.99	7.04	6.99	7.04	0.184	0.987
Lactic acid bacteria	8.41	8.32	8.41	8.47	8.35	0.121	0.961

SEM, standard error of the mean.

1) NC, negative control: basal diet; PC, positive control: NC+125 mg performance-enhancing antibiotic (enramycin 8%)/kg diet; EO100, NC+100 mg essential oil blend/kg diet; EO200, NC+200 mg essential oil blend/kg diet; EO400, NC+400 mg essential oil blend/kg diet.

**Table 6:** Effect of an essential oil blend on intestinal morphometry and morphology of weaning piglets at 42 days of experiment

Item	Treatments^1)^					SEM	p-value

	NC	PC	EO100	EO200	EO400		
Jejunum							
VH (mm)	0.34	0.32	0.38	0.32	0.36	0.011	0.329
CD (mm)	0.19	0.25	0.22	0.17	0.18	0.009	0.136
VH:CD	1.91	1.53	1.85	1.93	2.16	0.094	0.283
Infiltrate	1.17	1.33	1.17	1.17	1.33	-	0.762
Congestion	1.17	1.33	1.17	1.17	1.50	-	0.518
Desquamation	0.67	1.17	1.00	0.67	1.00	-	0.269
Necrosis	0.33	0.33	0.33	0.00	0.33	-	0.956
Edema	0.17	0.17	0.33	0.00	0.17	-	1.000
Ileum							
VH (mm)	0.36	0.32	0.29	0.34	0.36	0.014	0.304
CD (mm)	0.18	0.19	0.17	0.20	0.19	0.008	0.529
VH:CD	2.08	2.04	1.77	1.82	2.17	0.139	0.649
Infiltrate	0.67	1.33	1.33	1.00	1.00	-	0.115
Congestion	1.00	1.50	1.83	1.17	1.50	-	0.103
Desquamation	0.33	1.33	1.00	1.00	1.00	-	0.272
Bacterial lumps	0.00	0.00	0.17	0.17	0.33	-	1.000
Necrosis	0.00	0.17	0.00	0.00	0.00	-	0.307
Edema	0.00	0.17	0.17	0.17	0.50	-	0.143
Colon							
CD (mm)	0.36	0.41	0.37	0.33	0.33	0.024	0.537
Infiltrate	1.17	1.50	1.17	1.17	1.33	-	0.496
Congestion	1.33	1.00	1.17	1.17	1.33	-	0.398
Desquamation	0.00	0.33	0.67	0.67	0.67	-	0.406
Bacterial lumps	0.67	0.67	0.67	0.33	0.50	-	0.134
Edema	0.00	0.17	0.33	0.33	0.33	-	0.435

SEM, standard error of the mean; VH, villus height; CD, crypt depth.

1) NC, negative control: basal diet; PC, positive control: NC+125 mg performance-enhancing antibiotic (enramycin 8%)/kg diet; EO100, NC+100 mg essential oil blend/kg diet; EO200, NC+200 mg essential oil blend/kg diet; EO400, NC+400 mg essential oil blend/kg diet.

**Table 7:** Effect of an essential oil blend on relative weight and length of organs and pH of digestive contents of weaning piglets at 42 days of experiment

Item	Treatments^1)^					SEM	p-value

	NC	PC	EO100	EO200	EO400		
Relative weight (%)							
Stomach	0.77	0.73	0.80	0.79	0.76	0.010	0.249
Small intestine^2)^	4.56	4.18	4.13	4.10	4.37	0.101	0.425
Cecum	0.24	0.27	0.24	0.26	0.26	0.007	0.611
Large intestine^3)^	1.87	1.89	1.98	1.80	1.77	0.052	0.709
Heart	0.48	0.52	0.55	0.53	0.54	0.011	0.145
Liver and gallbladder	2.86	2.81	2.82	2.65	2.85	0.057	0.714
Kidneys	0.53	0.50	0.53	0.53	0.56	0.011	0.440
Spleen	0.23	0.23	0.25	0.24	0.23	0.006	0.903
Lenght (m)							
Small intestine^2)^	13.01	12.59	12.71	12.73	12.73	0.200	0.975
Large intestine^3)^	3.15	3.15	3.02	2.85	2.94	0.076	0.657
pH							
Stomach	3.10	4.34	3.58	3.63	4.07	0.204	0.181
Jejunum	5.90	5.20	5.13	6.08	5.77	0.204	0.355
Ileum	6.19	5.27	5.18	5.98	5.58	0.192	0.354
Cecum	5.31	5.00	5.07	5.51	5.38	0.094	0.358
Colon	5.37	5.68	5.82	5.69	5.81	0.075	0.087

SEM, standard error of the mean.

1) NC, negative control: basal diet; PC, positive control: NC+125 mg performance-enhancing antibiotic (enramycin 8%)/kg diet; EO100, NC+100 mg essential oil blend/kg diet; EO200, NC+200 mg essential oil blend/kg diet; EO400, NC+400 mg essential oil blend/kg diet.

2) Small intestine = duodenum+jejunum+ileum.

3) Large intestine = colon+rectum.

**Table 8:** Effect of an essential oil blend on the liver antioxidant status of weaning piglets at 42 days of experiment

Item	Treatments^1)^					SEM	p-value

	NC	PC	EO100	EO200	EO400		
GSH (μg/g of liver tissue)	747.70	673.21	732.15	718.55	719.85	12.984	0.450
GST (mmol/min/mg of LP)	17.55	17.38	19.24	16.68	17.83	0.811	0.862
SOD (U/mg of LP)	469.86^c^	615.20^a^	550.27^ab^	575.19^ab^	486.70^b^	18.940	0.047
CAT (mmol/min/mg of LP)	512.35	661.53	509.53	611.45	651.24	52.653	0.735
LP (mg/mL)	4,340	3,658	3,883	3,798	4,397	0.180	0.663

SEM, standard error of the mean; GSH, glutathione peptide; GST, glutathione S-transferase; SOD, superoxide dismutase; CAT, catalase; LP, liver protein.

1) NC, negative control: basal diet; PC, positive control: NC+125 mg performance-enhancing antibiotic (enramycin 8%)/kg diet; EO100, NC+100 mg essential oil blend/kg diet; EO200, NC+200 mg essential oil blend/kg diet; EO400, NC+400 mg essential oil blend/kg diet.

a–c Averages with different lowercase superscripts in the same row differ significantly by the Student-Newman-Keuls test (p<0.05).

## References

[1] Campbell JM, Crenshaw JD, Polo J (2013). The biological stress of early weaned piglets. J Anim Sci Biotechnol.

[2] Ma F, Xu S, Tang Z, Li Z, Zhang L (2021). Use of antimicrobials in food animals and impact of transmission of antimicrobial resistance on humans. Biosaf Health.

[3] Ngoh M, Wrzesinski C, Yang Y (2018). Evaluation of the presence and level of enramycin in broiler tissues following in-feed administration at therapeutic dose. J Appl Poult Res.

[4] Windisch W, Schedle K, Plitzner C, Kroismayr A (2008). Use of phytogenic products as feed additives for swine and poultry. J Anim Sci.

[5] Omonijo FA, Ni L, Gong J, Wang Q, Lahaye L, Yang C (2018). Essential oils as alternatives to antibiotics in swine production. Anim Nutr.

[6] Michiels J, Missotten J, Dierick N, Fremaut D, Maene P, De Smet S (2008). In vitro degradation and in vivo passage kinetics of carvacrol, thymol, eugenol and trans-cinnamaldehyde along the gastrointestinal tract of piglets. J Sci Food Agric.

[7] Rostagno HS, Albino LFT, Hannas MI (2017). Brazilian tables for poultry and swine: food composition and nutritional requirements.

[8] Nogueira ARA, Missaglia AP, Zani A, São Paulo SP (2017). Brazilian compendium of animal feed.

[9] Huang C, Qiao S, Li D, Piao X, Ren J (2004). Effects of lactobacilli on the performance, diarrhea incidence, vfa concentration and gastrointestinal microbial flora of weaning pigs. Asian-Australas J Anim Sci.

[10] da Silva N, Junqueira VCA, de Arruda Silveira NF, Taniwaki MH, Gomes RAR, Okazaki MM, São Paulo SP (2017). Manual of methods for microbiological analysis of food and water.

[11] Prophet EB, Mills B, Arrington JB, Sobin LH (1994). Laboratory methods in histotechnology.

[12] Kisielinski K, Willis S, Prescher A, Klosterhalfen B, Schumpelick V (2002). A simple new method to calculate small intestine absorptive surface in the rat. Clin Exp Med.

[13] Kraieski AL, Hayashi RM, Sanches A, Almeida GC, Santin E (2017). Effect of aflatoxin experimental ingestion and eimeira vaccine challenges on intestinal histopathology and immune cellular dynamic of broilers: applying an intestinal health index. Poult Sci.

[14] Aebi H (1984). Catalase in vitro. Methods Enzymol.

[15] Gao R, Yuan Z, Zhao Z, Gao X (1998). Mechanism of pyrogallol autoxidation and determination of superoxide dismutase enzyme activity. Bioelectrochem Bioenerg.

[16] Habig WH, Pabst MJ, Jakoby WB (1974). Glutathione s-transferases. The first enzymatic step in mercapturic acid formation. J Biol Chem.

[17] Sedlak J, Lindsay RH (1968). Estimation of total, protein-bound, and nonprotein sulfhydryl groups in tissue with Ellman's reagent. Anal Biochem.

[18] Bradford MM (1976). A rapid and sensitive method for the quantitation of microgram quantities of protein utilizing the principle of protein-dye binding. Anal Biochem.

[19] Kommera SK, Mateo RD, Neher FJ, Kim SW (2006). Phytobiotics and organic acids as potential alternatives to the use of antibiotics in nursery pig diets. Asian-Australas J Anim Sci.

[20] Tian Q, Piao X (2019). Essential oil blend could decrease diarrhea prevalence by improving antioxidative capability for weaned pigs. Animals.

[21] Li J, Wu XL, Chen Y (2013). Antidiarrheal properties of different extracts of Chinese herbal medicine formula Bao-Xie-Ning. J Integr Med.

[22] Arbati A, Maham M, Dalir-Naghadeh B (2021). The effect of cinnamaldehyde on the contractility of bovine isolated gastrointestinal smooth muscle preparations. Vet Res Forum.

[23] Barger AM (2003). The complete blood cell count: a powerful diagnostic tool. Vet Clin North Am Small Anim Pract.

[24] Hersey-Benner C, Mayer J, Donnelly TM (2013). Protein, total. Clinical veterinary advisor.

[25] Huang CM, Lee TT (2018). Immunomodulatory effects of phytogenics in chickens and pigs - a review. Asian-Australas J Anim Sci.

[26] Cavallazzi R, Bennin CL, Hirani A, Gilbert C, Marik PE (2010). Review of a large clinical series: is the band count useful in the diagnosis of infection? an accuracy study in critically ill patients. J Intensive Care Med.

[27] Schepetkin IA, Kushnarenko SV, Özek G (2016). Modulation of human neutrophil responses by the essential oils from Ferula akitschkensis and their constituents. J Agric Food Chem.

[28] Wei HK, Xue HX, Zhou ZX, Peng J (2017). A carvacrol–thymol blend decreased intestinal oxidative stress and influenced selected microbes without changing the messenger RNA levels of tight junction proteins in jejunal mucosa of weaning piglets. Animal.

[29] Gois FD, Cairo PLG, de Souza Cantarelli V (2016). Effect of brazilian red pepper (Schinus terebinthifolius Raddi) essential oil on performance, diarrhea and gut health of weanling pigs. Livest Sci.

[30] Cairo PLG, Gois FD, Sbardella M (2018). Effects of dietary supplementation of red pepper (Schinus terebinthifolius Raddi) essential oil on performance, small intestinal morphology and microbial counts of weanling pigs. J Sci Food Agric.

[31] Zhu LH, Zhao KL, Chen XL, Xu JX (2012). Impact of weaning and an antioxidant blend on intestinal barrier function and antioxidant status in pigs. J Anim Sci.

[32] Zou Y, Wang J, Peng J, Wei H (2016). Oregano essential oil induces SOD1 and GSH expression through Nrf2 activation and alleviates hydrogen peroxide-induced oxidative damage in IPEC-J2 cells. Oxid Med Cell Longev.

[33] Degroote J, Vergauwen H, Wang W, Van Ginneken C, De Smet S, Michiels J (2020). Changes of the glutathione redox system during the weaning transition in piglets, in relation to small intestinal morphology and barrier function. J Animal Sci Biotechnol.

[34] Asakura H, Kitahora T, Watson RR, Preedy VR, Zibadi S (2018). Chapter 23 - Antioxidants and polyphenols in inflammatory bowel disease: ulcerative colitis and crohn disease. Polyphenols: prevention and treatment of human disease.

[35] Ahsan U, Kuter E, Raza I (2018). Dietary supplementation of different levels of phytogenic feed additive in broiler diets: the dynamics of growth performance, caecal microbiota, and intestinal morphometry. Braz J Poult Sci.

